# A protocol to study the effect of targeted parental education intervention to identify early childhood development disorder – multisite interventional study

**DOI:** 10.1186/s13690-024-01495-y

**Published:** 2025-01-10

**Authors:** Vadivelan Kanniappan, Prakash Muthuperumal, P. Venkataraman, Anuradha Murugesan, Balaji Chinnasami, Manikumar Muthiah, Subramanian Sethuraman, Abishek J. R., Shrisruthi Suresh, Murali Krishnan Nambirajan, Angeline Grace G., Veeragoudhaman T. S., Kuberan Deivasigamani

**Affiliations:** 1 SRM College of Physiotherapy, Faculty of Medicine and Health Sciences, SRM Institute of Science and Technology, Chengalpattu, Kattankulathur 603203 India; 2 School of Public Health, Faculty of Medicine and Health Sciences, SRM Institute of Science and Technology, Chengalpattu, Kattankulathur 603203 India; 3 Department of Medical Research, Faculty of Medicine and Health Sciences, SRM Medical College Hospital and Research Centre, SRM Institute of Science and Technology, Chengalpattu, Kattankulathur 603203 India; 4 Department of Obstetrics and Gynaecology, Faculty of Medicine and Health Sciences, SRM Medical College Hospital and Research Centre, SRM Institute of Science and Technology, Chengalpattu, Kattankulathur 603203 India; 5 Department of Pediatrics, , Faculty of Medicine and Health Sciences, SRM Medical College Hospital and Research Centre,SRM Institute of Science and Technology, Chengalpattu, Kattankulathur 603203 India; 6 Smrthi Health Care, Chennai, 600004 India; 7 Public Health Consultant, Indian Council of Medical Research - National Institute of Epidemiology, Chennai, India; 8 Department of Community Medicine, Sree Balaji Medical College and Hospital, Chennai, 600044 India; 9 Community Medicine, ESIC Medical College & Hospital, K.K. Nagar, Chennai, 600078 India

**Keywords:** Neurological maturation, Development delay, Early interventions, Responsive parenting

## Abstract

**Background:**

In India, approximately 3.5 million children are affected by Developmental Delay (DD), often stemming from preterm births. These delays contribute to neurological and motor development delays, placing a significant financial burden on families. Maternal unemployment rates are also elevated in such cases. Delayed Developmental Milestones identification, often due to a lack of parental awareness, further compounds these challenges. This study introduces a multiphasic approach aimed at educating antenatal women on monitoring neurological maturation, with the overarching objective of assessing the effectiveness of a targeted multi-method parental education intervention in improving parents’ knowledge and early detection of developmental disorders in early childhood.

**Methods:**

Antenatal women will be assigned to intervention or control groups. The intervention group will undergo specialized training in a multiphasic study, while the control group will receive routine care. A meticulously developed intervention module for early detection of neurodevelopmental disorders will empower mothers to monitor their newborns for potential deficits. Outcomes will be assessed through questionnaires, analyzing knowledge improvement and early identification of DD using statistical methods such as chi-square tests. The study involves three phases: preparatory, implementation, and evaluation, aiming to empower mothers to detect developmental concerns early and improve maternal awareness of child development. The study was approved by the SRM Institutional Ethical Committee with the reference number 8688/IEC/2023.

**Discussion:**

This study will identify DD and improve parental awareness by providing tools for early detection thereby empowers parents to identify developmental concerns early. The study supports policy goals to reduce the burden of DD, enhance early intervention, and improve long-term outcomes for children. It is anticipated that this intervention will complement existing health policies, contributing to better child health and developmental outcomes in India.

**Trial registration:**

Trail is registered under Clinical Trails Registry - India (CTRI/2024/04/065008) registered on 01 April 2024.

**Table:** 

Text box 1. Contributions to the literature
• The utilization of multiphasic approaches such as scoping reviews, interviews, expert validation, co-designing, and pilot testing in the development of checklist and antenatal educational module enables iterative refinement.
• Educating mother right from the antenatal period about the red-flags in developments, helps increasing the rate of early screening and referral thereby mitigating the long term complications.
• In contrast to the common practice of adapting tools from a single established source, this research aims to develop a comprehensive tool that combines elements from multiple validated tools. This integrative approach ensures both methodological rigor and structural reliability.

## Background

Early childhood is a critical period for a child’s development. Developmental delays, if undetected and untreated, can have significant long-term consequences [1]. Developmental Delay (DD) pose a significant global health challenge, particularly affecting children in developing countries [2]. In South Asia, a staggering 53 million children under five are diagnosed with DD, with India bearing the brunt of this burden [3–5]. Globally, an estimated 180–200 million children under five experience developmental delays [6]. In India alone, at least 10% of young children are affected [7]. 

Preterm birth is one of the major contributors to neurodevelopmental disorders (NDDs) globally and in India. Studies indicate that one in two surviving preterm infants develops NDDs by the age of two. India contributes significantly to the global burden of preterm births, accounting for 23.4% of the total [8–11]. Premature infants often suffer from neurological impairments, such as incomplete myelination and immature brain development, which can lead to long-term developmental delays affecting their motor and cognitive abilities [12, 13]. Compounding this issue, factors like poverty, malnutrition, and limited access to healthcare further elevate the risk of DD [14]. Beyond the individual influence, DD have far-reaching consequences which impact multiple Sustainable Development Goals, including poverty, hunger, health, education, economic growth, and inequality [15]. Families of children with DD often face significant economic burdens, with 10% of mothers are forced to quit their jobs to care for their children [16]. In addition, socioeconomic factors such as poverty, malnutrition, and limited access to healthcare exacerbate the risk of DD [17].

Early identification of these delays is crucial for timely intervention and optimal outcomes [18, 19]. Parental awareness plays a key role in early detection, as parents are often the first to notice developmental deviations in their children [20]. However, a significant gap in maternal knowledge and awareness about developmental milestones and red flags persists, especially in rural and underserved regions in India, where access to healthcare resources is limited [21]. This lack of awareness will hinder the early identification and delay necessary interventions [22].

To address this pressing issue, it is crucial to empower mothers with the knowledge and skills to recognize early signs of developmental delays. Recognizing primitive reflexes and their persistence becomes a crucial diagnostic and prognostic factor for DD, especially during the pivotal 0–3 years when neurological maturity is at its peak [23–25]. Targeted parental education interventions provide a promising approach to empower parents to recognize developmental delays and seek timely treatment services [26]. By equipping parents with accurate information and practical tools, these interventions aim to enhance parental awareness, improve early identification rates, and ultimately optimize child development. By providing maternal education during the antenatal and postnatal periods, we can facilitate early diagnosis, prompt intervention, and ultimately improve the lives of children with DD [27, 28].

By implementing this strategy, we aim to facilitate early diagnosis, prompt specialized care, and improve long-term outcomes for children with developmental delays [29]. This will contribute to a brighter future where inclusion and healthy child development are prioritized [29]. Therefore, this study aims to evaluate the effectiveness of a targeted parental education intervention in improving parental awareness and early identification of developmental delays.

## Methods

### Research question

Will targeted parental education during the antenatal period about red flag signs in developmental milestones from 0 to 1 year improve early detection of childhood developmental disorders?

## Hypothesis

Mothers in the Intervention group can identify the early red flag signs of developmental disorders more easily than the control group.

### Study objectives

#### Primary objectives

1. To develop targeted multi-method parental education intervention on the detection of early red flag signs of early childhood developmental disorders.
2. To determine the effect of targeted multimethod parental education intervention on the identification of suspected early childhood developmental disorders by the parents.

#### Secondary objectives

1. To determine the yield of early detection by parents of early red flag signs of childhood developmental disorders.
2. To estimate the level of improvement of knowledge in early red flag signs of childhood development disorders among the parents.

### Methodology

This non randomized interventional study will be carried out among Antenatal mothers from 2nd Trimester of pregnancy at SRM Medical College Hospital, Research Centre, other established centres in Chennai and Trichy and Tanjavur Medical College, Tanjavur, Tamil Nadu, India. The study was approved by the Institutional Ethical Committee with the reference number 8688/IEC/2023 and was accepted by Indian Council of Medical Research (ICMR), a peer reviewed funding agency after reviewing for the methodological soundness. (Proposal id IIRP-2023-7817/F1). Trial has been registered under Clinical Trials Registry (CTRI/2024/04/065008).

### Eligibility criteria

For the intervention group, eligible participants include antenatal mothers who have provided informed consent and are willing to participate in the neuro-parenting program. These participants will need to be available for follow-up assessments at specified intervals to evaluate the intervention’s impact on early childhood development awareness. The control group will comprise antenatal mothers who are receiving routine antenatal and postnatal care without access to the neuro-parenting intervention; they will also provide informed consent and commit to follow-up visits. Exclusion criteria for both groups will include any mothers with medical conditions that could independently influence neurodevelopmental outcomes, as well as those who may face challenges in completing the follow-up.

**The study will be conducted in three phases namely**,

1. Preparatory Phase.
2. Implementation Phase.
3. Evaluation Phase.

#### Preparatory Phase

This phase will be a meticulous and iterative process, ensuring the development of a well-validated tool for assessing early indicators of neurodevelopmental disorders in infants which in turn, facilitates the effective monitoring and intervention strategies through parental education. Information on neurodevelopmental sequence, posture, milestones, reflexes will serve as the foundation for the material development. The following steps will be involved in this phase.

1. **Desk Review**: A comprehensive review of existing literature, encompassing both published and grey literature for identifying validated tools used to assess neuromotor maturity, neurodevelopmental outcomes, and indicators of neurodevelopmental disorders in infants.
2. **Expert Review**: Components will be gathered from the identified tools and will be charted according domains for each quarter of infant’s life. A panel discussion with domain experts, including paediatricians, neonatologists, physiotherapists, occupational therapists, speech language pathologists, developmental pediatricians, obstetricians, and gynaecologists will be conducted. Their valuable insights will guide the creation of a pilot version of the tool, incorporating critical assessment aspects and early indicators of developmental disorders detectable within the first year of life.
3. **Stakeholder Engagement**: Focus group discussions will be conducted among antenatal mothers, family members, community health workers and NICU staff nurses and efforts will be made to align the module standards with the local population’s knowledge level. Delphi rounds will be conducted to analyse the feasibility for the component to be taught to the mother and is safe for mother to assess. A co-design approach will be employed for the development of the tool. It also assesses module relevance, comprehensibility, and practicality, which will result in the refinement of the tool’s content.
4. **Pilot-testing**: The developed tool will be tested with a subset of targeted participants, which will provide valuable insights into practical challenges and real-world application. The outcomes will inform the feasibility of the tool and its alignment with the intended objectives.
5. **Enhancement of the Tool**: A feedback consultation with aforementioned stakeholders will cover various aspects, including implementation strategy, benefits, barriers, cost, comprehensibility, feasibility, and success rate. Collaborative adjustments, language and sociocultural adaptations for the refinement of the tool that will be comprehensible by any antenatal women or mother. The checklist will focus on all developmental domains, including primitive reflex, tone, fine motor skills, gross motor skills, cognitive, and social development.
6. **Formalization of the tool into an antenatal training module**: The refined material will be crafted to educate stakeholders on essential developmental concepts, emphasizing the significance of the first year of life, critical monitoring periods, checklist item explanations, assessment documentation, and interpretation. Antenatal counselling strategies are also included to alleviate parental stress.

A curriculum model will be developed for maternal education incorporating adult learning methods. Multimethod of teaching including video demonstration, in person teaching and Training of Trainers printed material-based teaching will be implemented.

Simultaneously, a thorough desk review is conducted to evaluate existing materials and videos aimed at enhancing mothers’ knowledge of early child development. Validity measures, including content validity, face validity, and construct validity, will be employed to ensure the robustness of the tool.

#### Implementation phase

**Fig. 1:**
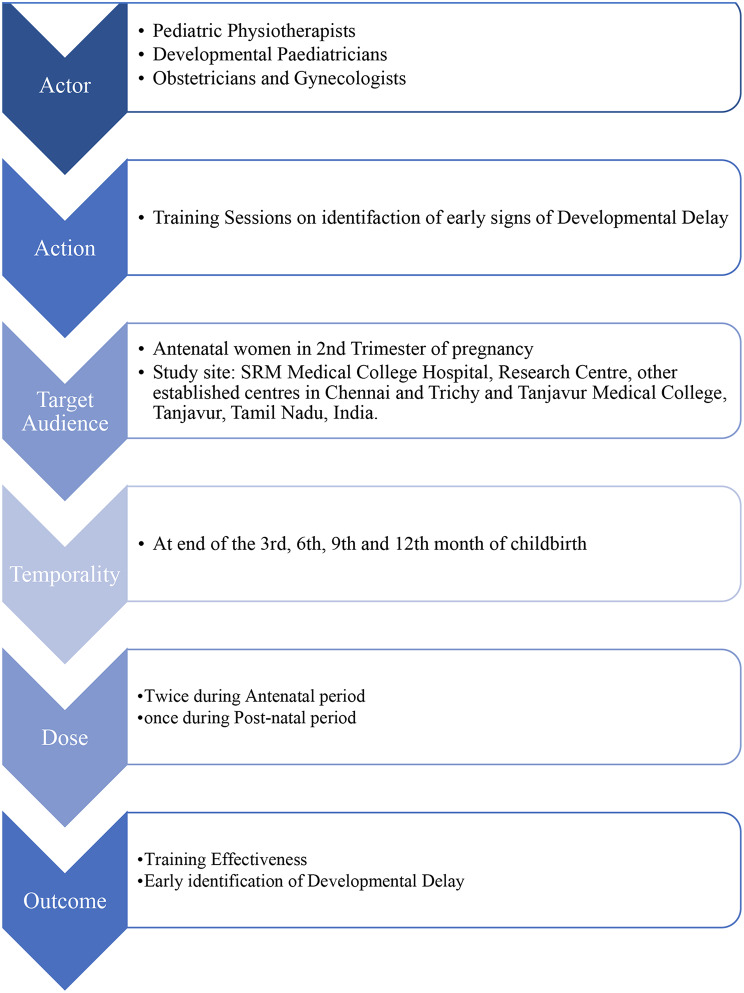
Implementation strategy

This Phase of the study employs a non-randomized intervention design to assess the impact of a targeted multimethod parental educational intervention on antenatal mothers. This phase seeks to provide valuable insights into the practical application and potential benefits of the neuro parenting program. A comparison is made between the intervention group, exposed to the neuro parenting program, and a control group receiving routine care without training sessions. The primary outcomes center around evaluating the percentage of mothers who suspect early childhood developmental disorders including true positive, false positive and false negative ratio. The confirmation of DD will be done using Bayley Scale for Infant Development IV. The study entails a meticulously structured follow-up period, with strategically defined time points to assess the program’s efficacy across critical stages of early development. Assessments will be conducted at birth, six months, and twelve months postpartum to monitor developmental trajectories. By demarcating these specific follow-up intervals, the study ensures a systematic framework for evaluating intervention outcomes, particularly the capacity of mothers to recognize neurodevelopmental indicators and suspect potential developmental disorders. This follow-up design is structured to capture the program’s lasting influence on maternal awareness and accuracy in identifying developmental milestones, offering a precise evaluation of the intervention’s long-term impact. Through targeted assessments, the study will measure both immediate and long-term effects on maternal ability to recognize early developmental cues, providing a focused understanding of the intervention’s effectiveness. Implementation strategies are illustrated in Fig. 1 and the timeline has been depicted in Table 1.

**Table 1:** Spirit time line

	Enrollment and Allocation					Post Allocation				Endline evaluation		Close Out	
**TIME POINTS**	-t4	-t3	-t2	-t1	t0	t1	t2	t3	t4	t5	t6	t7	t8
Team building	X	X											
Stake holder consultations and ethical proceedings	X	X											
Development of BCC Material			X	X									
Evaluation of BCC			X	X									
Preparation of questionnaire for baseline evaluation			X	X									
Baseline survey of knowledge of antenatal mothers					X								
Recruitment					X	X	X	X	X				
Training to antenatal women in intervention group (2 sessions)						X	X	X	X	X	X		
3rd training session – In postnatal period to intervention group								X	X	X	X		
Evaluation of training						X	X	X	X	X	X		
Interview with control mothers								X	X	X	X		
Endline evaluation of intervention								X	X	X	X		
Data analysis												X	X
Report writing												X	X

##### Sample size & sampling methodology

Anticipating a 5% exposure rate to neuro parenting among multigravida women during the study period and aiming for an odds ratio (OR) of 2 with 80% power and 95% confidence interval (CI), with 20% drop out rate the sample size calculation involves recruiting 513 participants per group, rounded to the nearest number, resulting in a total of 520 participants in each arm.

##### Intervention

Antenatal women with willingness to participate will be enrolled in the study after obtaining Informed Consent. The antenatal mothers who visit the hospital for antenatal care will be explained about the online module regarding its uses and accessibility of the information. In addition, they will be provided information regarding observing the growth and activities of the child. For the control group, antenatal mothers enrolled as controls, regular check ups as per the standard protocol and the printed material for identification of neurodevelopmental signs will be provided. The intervention comprises three strategically timed training sessions during the second trimester, third trimester and postnatal care, specifically targeting mothers of preterm babies.

#### Evaluation phase

This phase will assess the effectiveness of training program and the incidence of the DD among children born to mothers who participated in the training program. Mothers will have to undergo at least 2 of the three educational/training sessions.

### Training and assessment

In this phase, all participants will undergo a thorough assessment of training outcomes. The implementation strategy involves providing participants with a comprehensive questionnaire covering knowledge, awareness, and practices related to identifying early child development delays based on the training module. This questionnaire will be administered after the completion of the training module. Upon concluding all training sessions, a final questionnaire will be developed to gauge participants’ overall knowledge, practice, and understanding of the training’s importance. Descriptive statistics will be calculated to analyse quantitative variables, providing insights into the numerical aspects of the participants’ responses. For quantitative variables, frequencies and percentages will be computed, offering a quantitative overview of the participants’ understanding and practices. To explore the association between variables, the chi-square test will be employed, with a significance level set at 5%.

### Survey on neurodevelopmental outcomes

Targeting mothers who have attended parental educational interventions, a cross-sectional study will be employed to assess the percentage of mothers suspected of early childhood developmental disorders as a result of the training received.

### Interim and follow-up

Principal investigator will conduct interim analysis, regular follow ups to monitor the progress of the study and make final decision to terminate the trial.

During the follow-ups, participants will be asked to respond to a brief questionnaire addressing outcomes and early red flags identified. Descriptive statistics will then be employed to analyze the yield of early detection by parents alone, particularly focusing on red flags indicative of potential early childhood developmental disorders. This approach ensures a thorough evaluation of the impact of parental educational interventions on the early identification of developmental concerns among participating mothers.

The efficacy or productivity of the instruction is demonstrated through true positive results, as clarified in the outcome. True positives will be determined by comparing the evaluations conducted by parents with those carried out by healthcare professionals such as pediatricians and paediatric physical therapists.

### Evaluation of program effectiveness

At the end of all training sessions, a final questionnaire will assess how much knowledge, practical skill, and understanding participants gained from the program. It will focus on instances where mothers correctly identify developmental concerns, which are then confirmed by healthcare professionals. This evaluation will systematically measure the program’s effectiveness in equipping mothers with the ability to identify developmental disorders with precision. Beyond these direct impacts, the assessment will adopt a holistic approach, evaluating the intervention’s broader influence on multiple domains, including communication, learning, and behavioural development, to provide an in-depth understanding of its effectiveness in supporting comprehensive neurodevelopmental health.

## Discussion

The proposed intervention for developmental delay (DD) aims to create valuable outcomes for participants, the community, and the healthcare system [30]. By equipping parents with knowledge on early neuro-motor development tracking and red flag identification, the intervention has the potential to enable timely interventions, which may help to reduce the long-term impact of DD [31]. At the community level, home-based rehabilitation supported by mothers could promote inclusivity by integrating training modules into standard antenatal and postnatal care.

Additionally, the intervention may increase awareness and utilization of services like e-Sanjeevini, a National Teleconsultation Service [32]. 

The health system could also benefit from the development of a scalable screening tool, a comprehensive training manual for expectant mothers, and improved resources for healthcare professionals [30]. 

Future plans include integrating the program into routine check-ups, engaging community volunteers, and using tele-counselling technology to extend access. Furthermore, the commitment to generating unique and specialized resources reflects the potential to make a meaningful contribution to developmental paediatrics, supporting more effective and inclusive healthcare practices in the future.

## Data Availability

No datasets were generated or analysed during the current study.

## Data monitoring and trial decisions

All the data obtained in trial will be monitored by first author while routine monitoring will be done by the funding agency and investigators of the study. Any trial related decisions such as termination of trail will be taken by the first author/principal investigator.
